# Synthesis and in vitro antiproliferative activity of novel 12(*H*)-quino[3,4-*b*][1,4]benzothiazine derivatives

**DOI:** 10.1007/s00044-012-0384-4

**Published:** 2012-12-24

**Authors:** Andrzej Zięba, Małgorzata Latocha, Aleksander Sochanik

**Affiliations:** 1 Department of Organic Chemistry, Medical University of Silesia, 41-200 Sosnowiec, Poland; 2 Department of Cell Biology, Medical University of Silesia, 41-200 Sosnowiec, Poland; 3 Center for Translational Research and Molecular Biology of Cancer, Maria Skłodowska-Curie Memorial Cancer Center and Institute of Oncology, 44-100 Gliwice, Poland

**Keywords:** Phenothiazine, Azaphenothiazine, Quinobenzothiazine, Antiproliferative activity, Cisplatin

## Abstract

Novel method of N-dealkylating quinobenzothiazinium salts **2,** promoted by reaction with benzimidazole, led to a series of new azaphenothiazine derivatives having 12(*H*)-quino[3,4-*b*][1,4] benzothiazine **4** structure. Reaction of compounds **4** in an alkaline milieu with alkylating agents occur as *N*-alkylation of the thiazine nitrogen and yields quinobenzothiazine derivatives **7**. In vitro antiproliferative activity of compounds **4** and **7** was tested using two cancer cell lines (SNB-19 and C-32) and cisplatin as a reference. Most of the studied azaphenothiazine derivatives showed activity against both cell lines investigated (5.6–12.4 μg/ml concentration range tested). Compounds **4**(**b**–**e**) containing a halogen atom or methyl group at the 9-position of the quinobenzothiazine ring show activity in the tested concentration range only against C-32 cell line. Compound **4f** with methyl group in 11-position of quinobenzothiazine ring lacked activity against either cell line. The presence of additional aminoalkyl substituents at the thiazine nitrogen atom in compounds **7** increases their activity against both examined cell lines, when compared to compounds **4**.

## Introduction

Phenothiazines are an important class of three-ring heterocyclic compounds widely used in medicinal chemistry. Phenothiazines and their structural analogs (azaphenothiazines, benzophenothiazines) have been reported to possess antimicrobial (Bansode *et al.*, 2009; Klitgaard *et al.*, 2008), antitumor (Motohashi *et al.*, 2000, 2006; Pluta *et al.*, 2010), antioxidant (Kumar *et al.*, 2010; Morak-Młodawska *et al.*, 2010), antitubercular (Viveiros and Amaral, 2001; Amaral and Kristiansen, 2000), antimalarial (Dominguez *et al.*, 1997), antipsychotropic (Lin *et al.*, 1991; Isaacson, 1998), and anti-inflammatory (Sharma *et al.*, 2005) activities. Modification of basic structural fragments of drugs, by altering molecular conformation, introducing additional substituents into aromatic or heterocyclic rings can affect drug-receptor interactions, as well as drug body distribution and metabolism (Patrick, 2005). In our previous papers, we reported a novel method of synthesizing quinoline fragment-containing phenothiazine derivatives that possess the structure of 5-alkyl-12(*H*)-quino[3,4-*b*][1,4] benzothiazinium salts **2**. These compounds contain a totally planar tetracyclic fragment and have interesting antimicrobial and antiproliferative properties (Zięba *et al.*,^2010^, ^2012^). In this study, we present details of synthesis of novel quinobenzothiazine derivatives as free quinoline bases, and their derivatives containing aminoalkyl substituents at the thiazine nitrogen atom. We also demonstrate their antiproliferative activity.

## Results and discussion

### Chemistry

**Scheme. 1:**
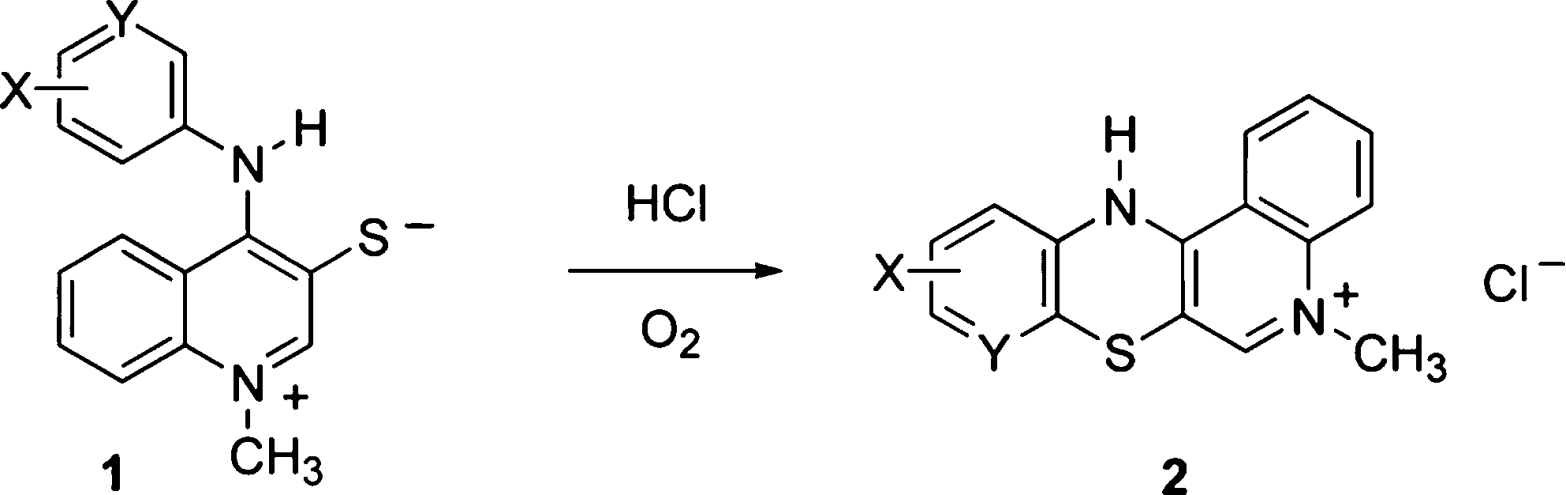
Synthesis of compounds **2**

5-Alkyl-12(*H*)-quino[3,4-*b*][1,4]benzothiazinium salts **2** were obtained by cyclization of 1-alkyl-4-(arylamino)quinolinium-3-thiolates **1** in the presence of HCl donor (aniline hydrochloride) and atmospheric oxygen (Scheme 1) (Zięba *et al.*, 2000; Zięba and Suwińska, 2006). 3-Thiolates **1** were obtained by reacting thioquinanthrenediinium salts with aromatic amines (Maślankiewicz and Zięba, 1992).

**Scheme. 2:**
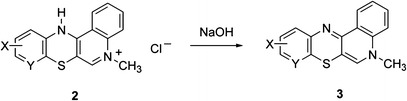
Reaction of salts **2** with bases

Phenothiazine derivatives with aminoalkyl substituents at the thiazine nitrogen atom constitute an important group of neuroleptic drugs (Isaacson, 1998), they also possess other interesting biological properties, such as antimicrobial and antiproliferative activity. Compounds having such structure are obtained by alkylating phenothiazine derivatives in an alkaline environment. Quinobenzothiazine derivatives with such substituents at the thiazine nitrogen atom cannot be obtained directly from salts **2** using this method, like 3-azaphenothiazine salts (Clarke *et al.*, 1961), they do not form sodium salts in the presence of bases. Instead, they split off hydrogen chloride and form respective 5-alkyl-5(*H*)-quino[3,4-*b*][1,4]benzothiazine **3** derivatives (Scheme 2) (Zięba *et al.*, 2000; Zięba and Suwińska, 2006).

**Scheme. 3:**
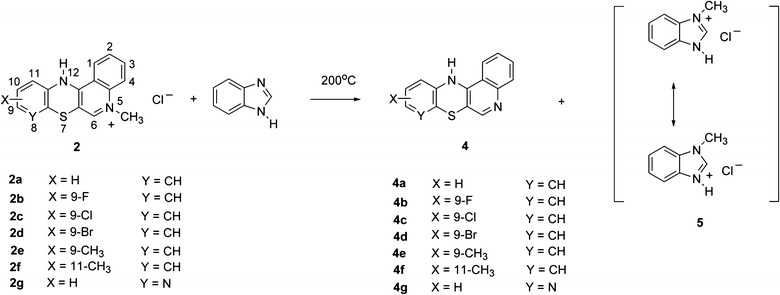
Synthesis of compounds **4**

We attempted, therefore, to perform N-dealkylation of salts **2** to obtain quinobenzothiazine derivatives **4** as free quinoline bases. There are no data available concerning N-dealkylation of azaphenothiazine salts. In an earlier publication, we described N-dealkylation of 1-alkylquinolinium salts achieved by heating their pyridine or DMF solutions (Maślankiewicz and Zięba, 1994). However, under such conditions salts **2** do not undergo the N-dealkylation reaction. On the other hand, by carrying the reaction of 5-alkyl-12(*H*)-quino[3,4-*b*][1,4]benzothiazinium salts **2** with benzimidazole at 200 °C, the expected 12(*H*)-quino[3,4-*b*][1,4]benzothiazines **4** were obtained (Scheme 3) with good yield. This reaction is a novel, so far unreported, method of N-dealkylating azaphenothiazine salts. The best results were obtained using a fivefold molar excess of benzimidazole with respect to quinobenzothiazinium salts **2**. It may be assumed that the other reaction product are benzimidazolium salts **5**, the structure of which can be stabilized via delocalization of positive charge among the benzimidazole nitrogen atoms.

Benzimidazolium salts **5** were neither isolated from the reaction mixture nor identified in the course of this study, as the primary objective here was to obtain quinobenzothiazine **4** derivatives as free quinoline bases. Excess benzimidazole and benzimidazolium salts **5** that form during the reaction were separated from quinobenzothiazines **4** by pouring post-reaction mixtures into water. Both benzimidazole and salts **5** are well-soluble in water, whereas compounds **4** fall out of solution as solids.

**Scheme. 4:**
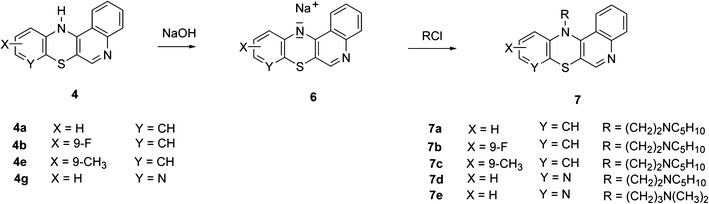
Synthesis of compounds **7**

**Scheme. 5:**
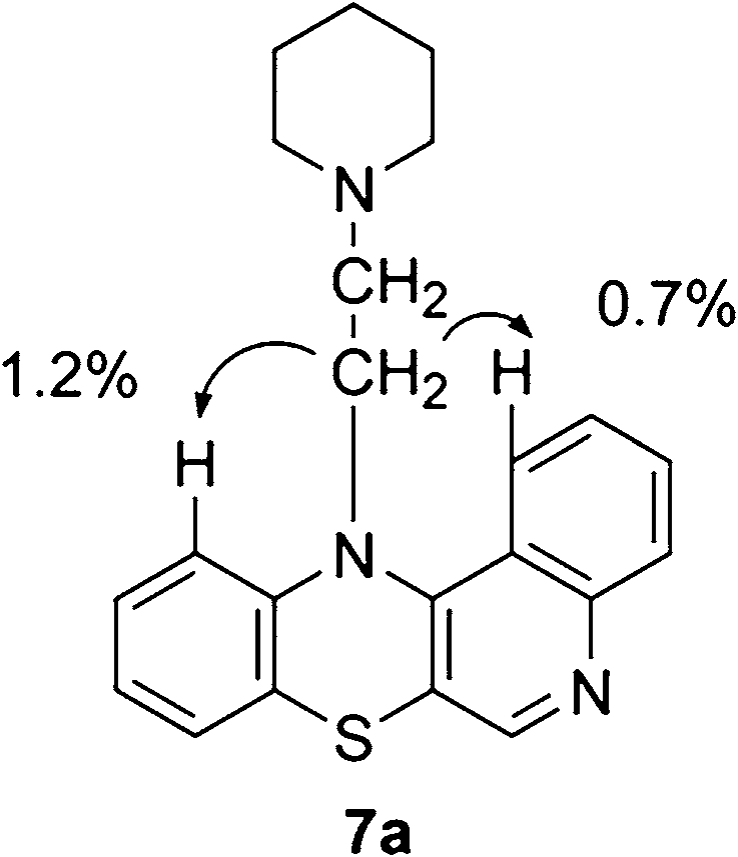
NOE ^1^H–^1^H homonuclear experiment for compound **7a**

In order to obtain quinobenzothiazine derivatives **7** containing aminoalkyl substituents at the thiazine nitrogen atom, compounds **4** were transformed, in the presence of sodium hydroxide, into salts **6**, which were then alkylated using aminoalkyl chlorides (Scheme 4). The reaction occurred as N-alkylation at the thiazine nitrogen atom and led to compounds **7**. The structure of compounds **7** was confirmed with ^1^H NMR spectroscopy by performing NOE ^1^H–^1^H homonuclear experiment. By irradiating methylene group protons at the thiazine nitrogen atom an enhancement of H1 and H11 proton signals from compounds **7** was obtained (Scheme 5).

### Antiproliferative activity

The activity of the obtained compounds **4** and **7** was investigated in vitro using cultured SNB-19 and C-32 cell lines and cisplatin as a reference. The examined quinobenzothiazines **4** had various substituents (CH_3_, F, Cl, Br) introduced into 9- and 11-positions of the quinobenzothiazine ring.

In addition, they also contain a nitrogen atom in the 8-position of the quinobenzothiazine ring.

Compounds **7** contains aminoalkyl substituents: 2-(*N*-piperidyl)ethyl (compounds **7**(**a–d**)) and 3-(*N*,*N*-dimethylamino)propyl **(**compound **7e**) at the thiazine nitrogen atom.

One of the mechanisms involved in antiproliferative effects of chemotherapeutics is DNA intercalation. This mode of action is typical for antiproliferative anthracycline antibiotics (e.g., doxorubicin) that feature planar tetracyclic (aromatic or heteroaromatic) fused rings. This mode of action, affecting cancer cells’ DNA, has been indeed suggested in reports concerning antiproliferative properties of phenothiazine and benzo[a]phenothiazine derivatives (Motohashi *et al.*, 2000; Hossain *et al.*, 2008; Hossain and Kumar, 2009). Structurally, compounds **4** and **7** studied herein are their analogs. The experiments demonstrated that the majority of the investigated compounds **4** and **7** showed antiproliferative activity toward examined cell lines within the 5.6–12.4 μg/ml concentration range (Table 1). In the case of compounds **4** (in the range of concentrations examined), the activity against both cell lines tested was displayed by compound **4a** which contains no additional substituents in the benzene ring, and compound **4g** which has an additional nitrogen atom at the 8-position of the quinobenzothiazine ring. Either compound showed similar activity against both cell lines. Such results may suggest that this structural fragment is not a decisive factor in antiproliferative activity of quinobenzothiazines **4** against SNB-19 and C-32 cell lines in vitro. Compounds **4**(**b**–**e**) containing a halogen atom or methyl group at the 9-position of the quinobenzothiazine ring show activity in the tested concentration range only against C-32 cell line. Compound **4f** with methyl group at the 11-position of the quinobenzothiazine ring did not display any activity against either cell line tested. The presence of additional aminoalkyl substituents at the thiazine nitrogen atom in compounds **7** increases their activity against both examined cell lines, when compared to compounds **4**.

**Table 1:** Antiproliferative activity in vitro of 12(*H*)-quino[3,4-*b*][1,4]benzothiazines **4**, **7** and cisplatin (reference) against two cancer cell lines studied

Compound	Antiproliferative activity IC_50_ (μg/ml)	
	SNB-19	C-32
**4a**	9.6 ± 0.9	8.9 ± 0.5
**4b**	Neg	9.4 ± 0.9
**4c**	Neg	7.8 ± 0.3
**4d**	Neg	8.6 ± 0.6
**4e**	Neg	8.7 ± 0.8
**4f**	Neg	Neg
**4g**	10.2 ± 0.6	8.7 ± 0.3
**7a**	6.7 ± 0.5	5.6 ± 0.4
**7b**	12.4 ± 1.2	7.0 ± 0.5
**7c**	6.6 ± 0.4	6.9 ± 0.8
**7d**	7.3 ± 0.7	7.9 ± 0.7
**7e**	8.2 ± 0.8	6.5 ± 0.5
Cisplatin	2.7 ± 0.3	5.8 ± 0.4

*Neg* negative at the concentration used

The results obtained herein demonstrate that replacement of aminoalkyl substituent, which contains a piperidyl ring, with a substituent containing *N*,*N*-dimethylamine group does not affect substantially antiproliferative activity. Compounds **7d** and **7e** which feature the same quinobenzothiazine ring but different aminoalkyl substituents at the nitrogen atom (12-position) show similar activity.

## Experimental

Melting points were determined in open capillary tubes and are uncorrected. NMR spectra were recorded using a Bruker DRX 500 spectrometer. Standard experimental conditions and standard Bruker program were used. The ^1^H NMR spectral data are given relative to the TMS signal at 0.0 ppm. EI MS spectra were recorded using an LKB GC MS 20091 spectrometer at 70 eV.

### Synthesis of 12(*H*)-quino[3,4-*b*][1,4]benzothiazines **4**

Mixture of 1 mmol quinobenzothiazinium salt **2** and 5 mmol (0.595 g) benzimidazole was heated for 2 h at 200 °C. The resulting reaction mix was dissolved in 10 ml ethanol and poured into 200 ml of water. The precipitate which formed was filtered off, washed with water, and air-dried. The raw product was purified by liquid chromatography using a silica gel-filled column and chloroform/ethanol (10:1 v/v) as eluent.

#### 12(H)-Quino[3,4-b][1,4]benzothiazine (***4a***)

Yield 79 %; m.p.: 204–205 °C; ^1^H-NMR (CD_3_OD, 500 MHz) *δ* (ppm): 6.85–6.91 (m, 2H, H_arom_), 6.93–6.97 (m, 1H, H_arom_), 6.99–7.04 (m, 1H, H_arom_), 7.48–7.52 (m, 1H, H-2), 7.59–7.63 (m, 1H, H-3), 7.76–7.80 (m, 1H, H-4), 8.07 (s, 1H, H-6), 8.12–8.16 (m, 1H, H-1); EI-MS *m*/*z*: 250 (M^+^, 100 %); Anal. calcd. for C_15_H_10_N_2_S: C, 71.97; H, 4.03; N, 11.19; S, 12.81. Found: C, 71.85; H, 3.97; N, 11.10; S, 12.77.

#### 9-Fluoro-12(H)-quino[3,4-b][1,4]benzothiazine (**4b**)

Yield 68 %; m.p.: 168–169 °C; ^1^H NMR (CD_3_OD, 500 MHz) *δ* (ppm): 6.64–6.68 (m, 1H, H_arom_), 6.70–6.75 (m, 1H, H_arom_), 6.87–6.91 (m, 1H, H_arom_), 7.44–7.49 (m, 1H, H-2), 7.56–7.61 (m, 1H, H-3), 7.73–7.76 (m, 1H, H-4), 8.01 (s, 1H, H-6), 8.05–8.09 (m, 1H, H-1); EI-MS *m*/*z*: 268 (M^+^, 100 %); Anal. calcd. for C_15_H_9_FN_2_S: C, 67.15; H, 3.38; N, 10.44; S, 11.95. Found: C, 67.09; H, 3.31; N, 10.40; S, 11.89.

#### 9-Chloro-12(H)-quino[3,4-b][1,4]benzothiazine (**4c**)

Yield 64 %; m.p.: 173–174 °C; ^1^H NMR (CD_3_OD, 500 MHz) *δ* (ppm): 6.88–6.91 (m, 2H, H_arom_), 7.02–7.05 (m, 1H, H_arom_), 7.55–7.60 (m, 1H, H-2), 7.68–7.73 (m, 1H, H-3), 7.78–7.82 (m, 1H, H-4), 8.12 (s, 1H, H-6), 8.17–8.20 (m, 1H, H-1); EI-MS *m*/*z*: 285 (M^+^, 100 %); Anal. calcd. for C_15_H_9_ClN_2_S: C, 63.27; H, 3.19; N, 9.84; S, 11.26. Found: C, 63.22; H, 3.15; N, 9.77; S, 11.23.

#### 9-Bromo-12(H)-quino[3,4-b][1,4]benzothiazine (**4d**)

Yield 54 %; m.p.: 96–98 °C; ^1^H NMR (CD_3_OD, 500 MHz) *δ* (ppm): 6.83–6.86 (m, 1H, H_arom_), 7.03–7.05 (m, 1H, H_arom_), 7.12–7.15 (m, 1H, H_arom)_, 7.48–7.54 (m, 1H, H-2), 7.60–7.66 (m, 1H, H-3), 7.77–7.81 (m, 1H, H-4), 8.06 (s, 1H, H-6), 8.09–8.14 (m, 1H, H-1); EI-MS *m*/*z*: 329 (M^+^, 100 %); Anal. calcd. for C_15_H_9_BrN_2_S: C, 54.73; H, 2.76; N, 8.51; S, 9.74. Found: C, 54.68; H, 2.73; N, 8.44; S, 9.71.

#### 9-Methyl-12(H)-quino[3,4-b][1,4]benzothiazine (**4e**)

Yield 83 %; m.p.: 202–203 °C; ^1^H NMR (CD_3_OD, 500 MHz) δ (ppm): 2.19 (s, 3H, CH_3_), 6.74–6.77 (m, 1H, H_arom_), 6.84–6.88 (m, 2H, H_arom_), 7.50–7.54 (m, 1H, H-2), 7.61–7.65 (m, 1H, H-3), 7.78–7.81 (m, 1H, H-4), 8.09 (s, 1H, H-6), 8.14–8.18 (m, 1H, H-1); EI-MS m/z: 264 (M^+^, 100 %); Anal. calcd. for C_16_H_12_N_2_S: C, 72.70; H, 4.58; N, 10.60; S, 12.13. Found: C, 72.61; H, 4.53; N, 10.53; S, 12.09.

#### 11-Methyl-12(H)-quino[3,4-b][1,4]benzothiazine (**4f**)

Yield 65 %; m.p.: 81–83 °C; ^1^H NMR (CD_3_OD, 500 MHz) *δ* (ppm): 2.36 (s, 3H, CH_3_), 6.77–6.84 (m, 2H, H_arom_), 6.90–6.95 (m, 1H, H_arom_), 7.50–7.55 (m, 1H, H-2), 7.59–7.64 (m, 1H, H-3), 7.70–7.82 (m, 1H, H-4), 7.98–8.03 (m, 1H, H-1), 8.13 (s, 1H, H-6); EI-MS *m*/*z*: 264 (M^+^, 100 %); Anal. calcd. for C_16_H_12_N_2_S: C, 72.70; H, 4.58; N, 10.60; S, 12.13. Found: C, 72.64; H, 4.55; N, 10.56; S, 12.09.

#### 12(H)-Pyrido[2,3-e]quino[3,4-b][1,4]thiazine (**4g**)

Yield 65 %; m.p.: 210–211 °C; ^1^H NMR (CD_3_OD, 500 MHz) *δ* (ppm): 6.97–7.01 (d.d, ^3^J = 8 Hz, ^3^J = 4.6 Hz, 1H, H-10), 7.67–7.90 (d.d, ^3^J = 8 Hz, ^4^J = 1.5 Hz, 1H, H_arom_), 7.51–7.55 (m, 1H, H-2), 7.62–7.67 (m, 1H, H-3), 7.77–7.81 (m, 1H, H-4), 7.84–7.86 (d.d, ^3^J = 4.6 Hz, ^4^J = 1.5 Hz, 1H, H_arom_), 8.07–8.11 (m, 2H, H-1, H-6)); EI-MS *m*/*z*: 251 (M^+^, 100 %); Anal. calcd. for C_14_H_9_N_3_S: C, 66.91; H, 3.61; N, 16.72; S, 12.76. Found: C, 66.86; H, 3.55; N, 16.69; S, 12.71.

### Synthesis of 12(H)-quino[3,4-*b*][1,4]benzothiazines **7**

A mixture of 15 ml water-free 1,4-dioxane, 1 mmol quinobenzothiazine **4** and 5 mmol (0.2 g) sodium hydroxide was refluxed for 2 h. Next, 10 ml of anhydrous benzene was added and the benzene-water azeotrope was distilled off. The resulting reaction mix was refluxed for 2 h while adding portionwise a 1.3 mmol aliquot of the alkylating factor (*N*-(3-chloropropyl)-*N*,*N*-dimethylamine hydrochloride or *N*-(2-chloroethyl)-piperidine hydrochloride). After cooling down to rt, the reaction mix was poured into 50 ml of water and extracted with 15 ml chloroform. The resulting solution was dried over anhydrous calcium chloride and evaporated under vacuum. The dry residue was purified by chromatography using a silica gel-filled column and chloroform-ethanol (10:1 v/v) as eluent. Quinobenzothiazines **7** were obtained as yellow oils.

#### 12-(2-(N-piperidyl)ethyl)-12(H)-quino[3,4-b][1,4]benzothiazine (**7a**) 

Yield 45 %; an oil; ^1^H NMR (CDCl_3_, 500 MHz) *δ* (ppm): 1.10-1.19 (m, 6H, H_piperidyl_), 2.05–2.18 (m, 4H, H_piperidyl_), 2.35–2.47 (t, J = 6.6 Hz, 2H, N_piperidyl_CH_2_), 4.12–4.28 (t, J = 6.6 Hz, 2H, CH_2_), 7.04–7.09 (m, 1H, H_arom_), 7.16–7.20 (m, 1H, H-11), 7.26–7.29 (m, 1H, H_arom_), 7.35–7.38 (m, 1H, H_arom_), 7.58–7.60 (m, 1H, H_arom_), 7.66–7.68 (m, 1H, H_arom_), 7.94–7.96 (m, 1H, H_arom_), 8.08–8.11 (m, 1H, H-1), 8.49 (s, 1H, H-6); EI-MS *m*/*z*: 361 (M^+^, 100 %); Anal. calcd. for C_22_H_23_N_3_S: C, 73.10; H, 6.41; N, 11.62; S, 8.87. Found: C, 73.11; H, 6.33; N, 11.56; S, 8.83.

#### 9-Fluoro-12-(2-(N-piperidyl)ethyl)-12(H)-quino[3,4-b][1,4]benzothiazine (**7b**)

Yield 56 %; an oil; ^1^H NMR (CDCl_3_, 500 MHz) δ (ppm): 1.22–1.42 (m, 6H, H_piperidyl_), 2.18–2.35 (m, 4H, H_piperidyl_), 2.48–2.67 (t, J = 7.1 Hz, 2H, N_piperidyl_CH_2_), 4.12–4.24 (t, J = 7.1 Hz, 2H, CH_2_), 6.85–6.88 (m, 1H, H-8), 6.89–6.95 (m, 1H, H-10), 7.12–7.18 (m, 1H, H-11), 7.48–7.54 (m, 1H, H-2), 7.58–7.64 (m, 1H, H-3), 7.98–8.04 (m, 2H, H-1, H-4), 8.48 (s, 1H, H-6); EI-MS *m*/*z*: 379 (M^+^, 100 %); Anal. calcd. for C_22_H_22_FN_3_S: C, 69.63; H, 5.84; N, 11.07; S, 8.45. Found: C, 69.51; H, 5.79; N, 11.00; S, 8.41.

#### 9-Methyl-12-(2-(N-piperidyl)ethyl)-12(H)-quino[3,4-b][1,4]benzothiazine (**7c**)

Yield 52 %; an oil; ^1^H NMR (CDCl_3_, 500 MHz) δ (ppm): 1.24–1.43 (m, 6H, H_piperidyl_), 2.20–2.34 (m, 7H, CH_3_, H_piperidyl_), 2.54–2.61 (t, J = 7.3 Hz, 2H, N_piperidyl_CH_2_), 4.17–4.23 (t, J = 7.3 Hz, 2H, CH_2_), 6.92–6.97 (d, ^4^J = 1.1 Hz, 1H, H-8), 6.98–7.02 (d.d, ^3^J = 8.2 Hz, ^4^J = 1.1 Hz, 1H, H-10), 7.06–7.09 (d, ^3^J = 8.2 Hz, 1H, H-11), 7.46–7.51 (m, 1H, H-2), 7.57–7.62 (m, 1H, H-3), 7.98–8.0 (m, 2H, H-1,H-4)), 8.48 (s, 1H, H-6); EI-MS *m*/*z*: 376 (M^+^, 100 %); Anal. calcd for C_23_H_25_N_3_S: C, 73.56; H, 6.71; N, 11.19; S, 8.54. Found: C, 73.50; H, 6.64; N, 11.12; S, 8.48.

#### 12-(2-(N-piperidyl)ethyl)-12(H)-pyrido[2,4-e]quino[3,4-b][1,4]thiazine (**7d**) 

Yield 49 %; an oil; ^1^H NMR (CDCl_3_, 500 MHz) δ (ppm): 1.22–1.32 (m, 6H, H_piperidyl_), 2.01–2.28 (m, 4H, H_piperidyl_), 2.41–2.50 (t, J = 6.6 Hz, 2H, N_piperidyl_CH_2_), 4.01–4.12 (t, J = 6.6 Hz, 2H, CH_2_), 7.02–7.08 (m, 1H, H-11), 7.28–7.34 (m, 1H, H_arom_), 7.41–7.47 (m, 1H, H_arom_),7.52–7.59 (m, 1H, H_arom_), 7.92–7.99 (m, 2H, H_arom_), 8.06–8.11 (m, 1H, H-1), 8.44 (s, 1H, H-6); EI-MS *m*/*z*: 362 (M^+^, 100 %); Anal. calcd. for C_21_H_22_N_4_S: C, 69.58; H, 6.12; N, 15.46; S, 8.84. Found: C, 69.54; H, 6.07; N, 15.40; S, 8.82.

#### 12-(3-(N,N-dimethylamino)propyl)-12(H)-pyrido[2,4-e]quino[3,4-b][1,4]thiazine (**7e**)

Yield 58 %; an oil; ^1^H NMR (CDCl_3_, 500 MHz) δ (ppm): 1.63–1.78 (m, 2H, CH_2_ CH_2_CH_2_), 1,98 (s, 6H, N(CH_3_)_2_), 2.18–2.24 (t, J = 7.2 Hz, 2H, (CH_3_)_2_NCH_2_), 4.01–4.12 (t, J = 7.3 Hz, 2H, NCH_2_), 7.04–7.11 (m, 1H, H-11), 7.28–7.36 (m, 1H, H_arom_),7.41–7.48 (m, 1H, H_arom_), 7.53–7.61 (m, 1H, H_arom_), 7.98-8.01 (m, 2H, H_arom_), 8.08–8.14 (m, 1H, H-1), 8.46 (s, 1H, H-6); EI-MS *m*/*z*: 336 (M^+^, 100 %); Anal. calcd. for C_19_H_20_N_4_S: C, 67.83; H, 5.99; N, 16.65; S, 9.53. Found: C, 67.74; H, 5.93; N, 16.61; S, 9.50.

### Antiproliferative assay in vitro

#### Cell culture

The synthesized compounds were evaluated for their anticancer activity using two cultured cell lines: SNB-19 (human glioblastoma, DSMZ - German Collection of Microorganisms and Cell Cultures, Braunschweig, Germany) and C 32 (human amelanotic melanoma, ATCC—American Type Culture Collection, Rockville, MD, USA). The cultured cells were kept at 37 °C and 5 % CO_2_. The cells were seeded (1 × 10^4^ cells/well/100 μl D-MEM supplemented with 12 % FCS and streptomycin and penicillin) using 96-well plates (Corning).

#### WST-1 assay

Antiproliferative effect of compounds **4** and **7** was determined using the Cell Proliferation Reagent WST-1 assay (Roche Diagnostics, Mannheim, Germany). This colorimetric assay is based on the cleavage of the tetrazolium salt WST-1 by mitochondrial dehydrogenases in viable cells, leading to formazan formation. After exposure to tested compounds (at concentrations between 0 and 100 μg/ml) for 72 h, cells were incubated with WST-1 (10 μl) for 2 h, and the absorbance of the samples against a background control was read at 450 nm using a microplate reader. Results are expressed as means of at least two independent experiments performed in triplicate.
